# SARS-CoV-2 Leads to Significantly More Severe Olfactory Loss than Other Seasonal Cold Viruses

**DOI:** 10.3390/life12030461

**Published:** 2022-03-21

**Authors:** Antje Haehner, Belinda Marquardt, Romina Kardashi, Katja de With, Susann Rößler, Basile Nicolas Landis, Antje Welge-Luessen, Thomas Hummel

**Affiliations:** 1 Smell & Taste Clinic, Department of Otorhinolaryngology, TU Dresden, 01069 Dresden, Germany; belinda.marquardt@mailbox.tu-dresden.de (B.M.); thomas.hummel@tu-dresden.de (T.H.); 2 Division of Infectious Diseases, TU Dresden, 01307 Dresden, Germany; romina.kardashi@uniklinikum-dresden.de (R.K.); katja.dewith@uniklinikum-dresden.de (K.d.W.); susann.roessler@uniklinikum-dresden.de (S.R.); 3 Institute of Medical Microbiology and Virology, TU Dresden, 01307 Dresden, Germany; 4 Rhinology-Olfactology Unit, Department of Otorhinolaryngology-Head and Neck Surgery, University Hospital of Geneva Medical School, 1205 Geneva, Switzerland; basile.landis@hcuge.ch; 5 Department of Otorhinolaryngology, University Hospital Basel, 4051 Basel, Switzerland; antje.welge-luessen@usb.ch

**Keywords:** COVID-19, SARS-CoV-2, smell, olfactory loss, virus

## Abstract

The aim of this study was to investigate whether COVID-associated olfactory impairment differs from olfactory disorders due to other upper respiratory tract infections. We investigated the frequency of a SARS-CoV-2 infection among subjects presenting with a subjective olfactory impairment to a corona outpatient clinic between October 2020 and March 2021. Olfactory and gustatory loss were tested psychophysically, and the type of infection, SARS-CoV-2 versus 14 other common cold viruses, was assessed with nasopharyngeal swabs. Differences between the smell impairment caused by the pathogens were compared. Out of the 2120 patients, 314 reported sudden smell and/or taste loss (14%). In 68.9% of them, olfactory and in 25.6%, gustatory dysfunction could be confirmed by psychophysical testing. Of those with a psychophysically determined loss of smell, 61% were tested positive for SARS-CoV-2. SARS-CoV-2 led to a significantly more severe loss of smell and more qualitative olfactory disorders than other pathogens. Apart from rhinorrhea, shortness of breath and sore throat accompanying cold symptoms do not differ significantly between the viruses indicating the particular importance of smell loss in the differential diagnosis of seasonal colds. Multiplex-PCR in non-COVID patients revealed that only 27% of them had rhinoviruses, whereas the remainder were no further identified pathogens. Olfactory screening significantly increases diagnostic accuracy in COVID-19 patients compared to subjective assessment of olfactory loss.

## 1. Introduction

Viral upper respiratory tract infections (URTI) are among the most common causes of olfactory loss. Considering that more than 200 viruses are known to cause URTIs, virus-specific differences in the frequency of subsequent olfactory disorders might be expected. Together with rhinoviruses, adeno-, influenza-, and parainfluenza viruses, coronaviruses have long been known to account for at least 70% of common colds [1]. Although available literature on this topic is sparse, olfactory disorders have also been reported in parainfluenza viruses [2,3], influenza virus [4], and rhinoviruses [5]. The frequency of coronavirus infections has never been a serious health issue [2,4,5] until the onset of the current corona pandemic. Smell and/or taste loss have been observed in up to 80% of these patients [6,7,8], sometimes as the only apparent symptom, at a younger age and a very early stage of the disease [9]. Several pathophysiologic mechanisms have been proposed for olfactory dysfunction due to SARS-CoV-2 infection. Recent research results provide evidence for the fact that sustentacular (non-neuronal) cells are the main target cell type in olfactory mucosa due to their expression of the receptor ACE2. Structural and/or physiological disruption of these cell populations may impair olfactory receptor neuron (ORN) function with no evidence for infection of ORNs or olfactory bulb parenchyma [10].

Since May 2020, the WHO has listed smell and taste disorders as a typical symptom of COVID-19, which enormously raised awareness of the chemosensory loss. Thus, people with a cold and new-onset smell and taste disorders have mainly presented themselves to corona outpatient clinics. This raises the question of how the loss of smell and taste might differ between the various causes of URTI. A few studies have already identified differences of COVID-related olfactory loss to smell disorders in influenza and in the context of acute common cold [11,12,13], suggesting a higher frequency of olfactory disorders in SARS-CoV-2 infection along with a more pronounced severity. This might indicate clinically relevant peculiarities in the case of COVID-associated olfactory loss.

The aim of this study was to investigate the frequency of a SARS-CoV-2 infection among those who presented to a university coronavirus testing center with smell loss and/or taste loss in winter 2020/2021, to verify the sensory loss with a reliable test and to examine possible differences between the smell impairment caused by SARS-CoV-2 and other viruses causing URTI. Further, the type of the virus should be determined in patients with smell and/or taste loss and a negative RT-PCR test.

## 2. Materials and Methods

Patients who presented to the coronavirus testing center at the University Hospital Dresden between October 2020 and February 2021 were routinely asked about new smell and taste disorders. Those with these symptoms received a *standardized diagnostic questionnaire* which included the patients’ main symptoms, time course, and an additional self-assessment of the patients’ current smell, taste function, and nasal breathing compared to the level before the onset of symptoms. The patients had to indicate whether they experienced loss of smell and/or taste (yes vs. no). For quantifying olfactory/gustatory function and nasal breathing, we used a visual analog scale (VAS) with its extreme left of the scale defined as “no function” (0 units), and the extreme right of the scale defined as the highest function possible (“extremely good“—10 units).

Further, a short *olfactory screening test* based on pen-like odor dispensing devices was applied [14]. This odor identification test contains 5 common odors to be all identified from a list of 20 choices. During the test, the examiner removes the cap and places the pen’s tip about 2 cm below the participants’ nose to release the corresponding odor. If necessary, participants can sniff multiple times to make a choice. Each pen is presented by the examiner with an interval of approximately 30 s to prevent olfactory desensitization. Each correct recognition of the odor scores 1 point, with the highest identification scores as 5. Hyposmia was considered if the score was between 1 and 3 and functional anosmia if it was 0.

For taste examination, four *taste strips* consisting of filter papers impregnated with the following 4 tastants [15]: sucrose (sweet), citric acid (sour), sodium chloride (salty), and quinine hydrochloride (bitter) were applied. After participants themselves placed one strip onto their own tongue, they closed their mouths and were allowed to suck the strip. Then they were forced to identify the taste from 4 possible options mentioned above. Participants were asked to take a sip of water before testing each strip. Hypogeusia was considered if the score was below 3.

For *virus detection*, specimens were collected via throat swabs and transported in liquid medium. The BioFire^®^ FilmArray^®^ Respiratory Panel 2 (Biomérieux, Marcy l’Etoile, France), a multiplex-PCR test system, was applied. This test is intended to detect simultaneously nucleic acids from the following 14 viruses (and 3 bacteria species) causing respiratory tract infections: adenovirus, coronavirus 229E/HKU1/NL63/OC43, human metapneumovirus, human rhinovirus/enterovirus, influenza virus A/B, parainfluenza virus 1–4, respiratory syncytial virus (RSV), *Bordetella pertussis*, *Chlamydia pneumoniae,* and mycoplasma pneumoniae. Briefly, 300 µL of the sample is used to prepare the sample mix, the following steps (cell lysis, extraction, amplification, detection) are performed in the closed pouch system of the array [16].

The *statistical analysis* was performed with SPSS (version 27, SPSS Inc., Chicago, IL, USA). If not mentioned otherwise, all data are displayed as means ± standard deviations (SD) or percentages (%). Results were submitted to two-sided paired *t*-tests, chi-square-tests, Mann–Whitney U-Tests, and analyses of variance. Bonferroni tests were used for post-hoc analyses. For all tests, the level of significance was set at α < 0.05.

## 3. Results

### 3.1. Study Population

Out of the 2120 patients (mean age, 34.4 years ± 14.8) who presented with symptoms of a common cold and fulfilled national SARS-CoV-2 testing criteria [17], 46.8% were male and 53.2% female. Main symptoms were rhinorrhea (58.9%), headache (58.0%), sore throat (55.7%), cough (55.1%), and myalgia (38.2%), followed by fever (13.9%), diarrhea (13.5%), dyspnea (10.4%), and nausea (10.4%). In 627 patients, SARS-CoV-2 infection was detected by a positive RT-PCR.

### 3.2. Subjective Smell and/or Taste Loss

Out of the 2120 patients, 314 (14.8%) reported sudden smell and/or taste loss. In 137 patients (*n* = 43.6%) with subjective chemosensory loss, the diagnosis of SARS-CoV-2 infection was confirmed by means of a positive RT-PCR test (Table 1), whereas 27.1% of the patients without chemosensory loss were tested positive (χ^2^ = 34.9, *p* < 0.001).

### 3.3. Psychophysical Testing of Smell and/or Taste Function

Out of the 314 patients with subjective chemosensory loss, 238 agreed to have a smell and taste test. In those cases, an olfactory dysfunction was verified in 164 patients (68.9%). Out of these patients with psychophysically determined olfactory loss, 100 (61%) were tested positive for SARS-CoV-2. On the other hand, only 17.6% of the patients without proven smell loss were tested positive for SARS-CoV-2 (χ^2^ = 38.5, *p* < 0.001) (Table 2).

A taste loss was verified in 64 individuals (25.6%). Out of these patients with psychophysically determined taste deficits, 27 (44.3%) were tested positive for SARS-CoV-2. On the other hand, 48.6% of the patients without proven taste loss were tested positive for SARS-CoV-2 (χ^2^ = 0.34, *p* = 0.66) (Table 3). In those with a proven combined smell and taste loss (*n* = 25), 56% were tested positive for SARS-CoV-2.

### 3.4. Identification of Pathogens Other than SARS-CoV-2

In 73 patients with subjective smell and/or taste loss and a negative SARS-CoV-2 RT-PCR test, a multiplex-PCR on throat samples was performed. In 20 of 73 samples, nucleic acid of rhinovirus/enterovirus (REV) was detected. Due to the genetic similarity of human rhinovirus and enterovirus, further differentiation using this multiplex-PCR system is not possible.

The presence of adenovirus, coronavirus 229E/HKU1/NL63/OC43, human metapneumovirus, influenza A/B, parainfluenza virus 1/2/3/4, and RSV was also investigated but was not detected in any of the samples. The pathogens of the other infections with olfactory and taste disorders could therefore not be identified and are unknown.

### 3.5. Comparison between Smell and Taste Loss Related to SARS-CoV-2 and Other Causes

**Figure 1:**
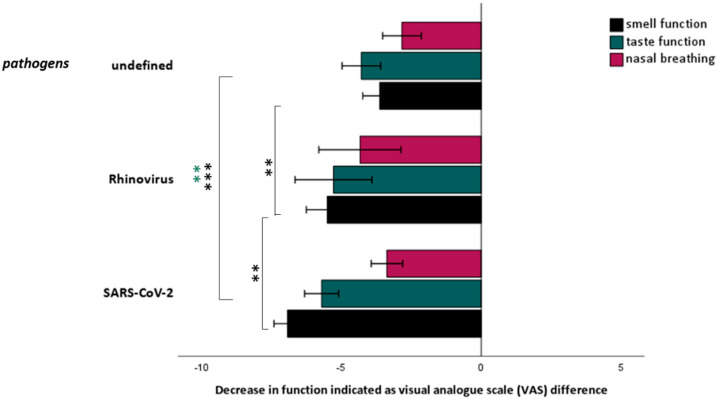
Decrease in smell/taste function and nasal breathing indicated as the difference between visual analog scale (VAS) ratings before the infection and during acute infection. The 10-point VAS was defined with its extreme left of the scale defined as “no function” (0 units), and the extreme right of the scale defined as the highest function possible (“extremely good“—10 units). In the “undefined” pathogens group presence of adenovirus, coronavirus 229E/HKU1/NL63/OC43, metapneumovirus, influenza A/B, parainfluenza virus 1/2/3/4, RSV, Bordetella pertussis, *Chlamydia pneumonia*, and *Mycoplasma pneumoniae* was investigated but was not detected. ** *p* < 0.01. *** *p* < 0.001.

No age (F = 1.03, *p* = 0.38) and sex differences (χ^2^ = 1.15, *p* = 0.77) emerged between the various causes of chemosensory loss (SARS-CoV-2, REV, undefined pathogens). SARS-CoV-2 and other causes related smell loss differed significantly in terms of severity of subjective (VAS score) (F = 80.1, *p* < 0.001) and psychophysically determined smell loss (Sniffin Stick score) (F = 93.1, *p* < 0.001) with post-hoc comparison revealing significant differences between SARS-CoV-2 and REV (*p* < 0.001) and unknown pathogens (*p* < 0.001) (Figure 1). While 59.8% of the patients with SARS-CoV-2-related smell loss reported complete loss of smell, this was mentioned in 16.7% of the REV and in 9.4% of the patients with undefined pathogens only. Qualitative smell changes (parosmia and phantosmia) were reported in 10.3% of the patients with SARS-CoV-2-related smell loss and in 5.7% of undefined pathogens, but not in REV (χ^2^ = 54.6, *p* < 0.001).

While severity of subjective taste loss between the groups differed significantly (VAS score) (F = 12.8, *p* < 0.001) (Figure 1) results from psychophysical testing (taste strips score) revealed no differences (F = 0.43, *p* = 0.51).

Nasal breathing was mostly impaired in REV, followed by SARS-CoV-2 and unknown pathogens. However, there was no significant differences between the 3 groups (F = 2.0, *p* = 0.11) (Figure 1).

Smell and taste loss mainly occurred after the onset of other symptoms in SARS-CoV-2 and REV, whereas it was noticed most often at the same time as other symptoms in undefined viruses (F = 9.1, *p* < 0.001).

With regard to the accompanying symptoms, significant differences were found between the virus groups for rhinorrhea, shortness of breath, and sore throat. Patients with REV infection presented more often with rhinorrhea and shortness of breath than SARS-CoV-2 patients or patients with undefined pathogens. A sore throat, however, was significantly more often associated with an undefined virus-associated smell loss compared to SARS-CoV-2 or REV. Among the symptoms asked for, coughing, rhinorrhea, shortness of breath, sore throat, fever, headache, nausea, diarrhea, muscle, and limb pain, only diarrhea was the most common with SARS-CoV-2 infection, although no significance was reached (F = 0.34, *p* = 0.77).

## 4. Discussion

In this large URTI patient cohort study of a coronavirus testing center, the following main results emerged. First, 14.8% of patients reported sudden smell and/or taste loss. In 68.9% of them, olfactory loss and in 25.6%, a taste loss was confirmed by testing. Second, by applying a psychophysical olfactory test to patients with subjective chemosensory disorders, the proportion of SARS-CoV-2-positive patients was increased from 44% to 61%. This change exceeds the benefit of a taste test or a combined smell and taste testing in terms of confirmation of the diagnosis. Third, the majority of non-SARS-CoV-2-related olfactory loss is caused by unknown pathogens. Rhino/Enteroviruses were responsible for the loss of smell in only 27%. Fourth, in the acute stage of infection, SARS-CoV-2 leads to a significantly more severe loss of smell and more qualitative olfactory disorders than other viruses. Further, apart from rhinorrhea, shortness of breath and sore throat as accompanying cold symptoms do not differ significantly between the pathogens.

Consistent with other studies, in COVID-19, we found a much higher incidence of olfactory loss than taste disorders using chemosensory testing [8,18]. This is in contrast to the subjective assessment of the patients and might be attributed to impaired retronasal olfaction (flavor) rather than impaired gustation (sweet, salty, sour, bitter, and umami). Therefore, study results point toward a primary olfactory deficit, which should ideally be confirmed with an olfactory test for which fast, inexpensive, and reliable screening methods are available (e.g., disposable Q-Sticks Tests [19]). Our results show that taste tests or combined olfactory/gustatory testing do not significantly contribute to the COVID-19 diagnosis; a simple olfactory test is definitely more sensitive. Some studies in the past came to a different conclusion and attached more importance to taste testing (e.g., the work of [20,21]. They used, however, improvised, self-administered home tests, which have several weaknesses regarding the isolated testing of taste ability without olfactory components.

In contrast to what we had expected in view of the available literature, the majority of the non-SARS-CoV-2-related loss of smell is caused by unknown pathogens. According to a comprehensive study of over 7600 patients, in addition to SARS-CoV-2, rhinoviruses, influenza viruses, other coronaviruses, respiratory syncytial viruses, metapneumoviruses, and parainfluenza viruses were mainly responsible for severe cold symptoms in the first pandemic winter 2019/20 [22]. Of these, rhinovirus, human coronavirus, and parainfluenza virus are known to cause olfactory dysfunction [2,3,5,23] and were expected to be the cause of the non-SARS-CoV-2 olfactory loss. However, REV could only be detected in 27%, while none of the known common cold viruses was positive for the remaining patients with non-SARS-CoV-2 smell loss. This raises the question to what extent the virus distribution in winter 2020/2021 differed from the previous one and to what extent our view of olfactory loss-associated viruses needs to be revised.

In our study, we found a pronounced severity of SARS-CoV-2-related smell loss compared to non-SARS-CoV-2-related olfactory loss. While the majority of the SARS-CoV-2 group reported a complete loss of smell, this applied to only 9%–16% for the other viruses, which confirmed results from other studies [12,24]. In general, qualitative olfactory disorders such as parosmias and phantosmias are rare during acute colds and tend to appear with a latency of several months. In COVID-19, however, parosmias have already been reported in early disease stages [6]. In line with this, chemosensory distortions in this study occurred at significantly higher rates in acute SARS-CoV-2 infections compared to other colds. Similar results have been reported in recent studies [25,26], but sometimes with much higher incidences [24] which might be explained by the time interval between the onset of the cold and survey completion. In our study, all patients were interviewed at the time of diagnosis. Further, the frequency of the cold symptoms cough, headache, fever, nausea, diarrhea, muscle and limb pain did not differ significantly between smell loss due to SARS-CoV-2, REV, or unknown viruses. Rhinorrhea and shortness of breath were significantly more common with REV infection, while sore throat was significantly most frequently mentioned with unknown pathogens. The fact that rhinorrhea rarely occurs with COVID-19 has already been described in other studies [8,27], although in the present study, there was no difference to the common cold caused by unknown viruses. This, in turn, suggests that the pronounced severity of the sudden loss of smell might be the most typical sign of a SARS-CoV-2 infection compared to other colds.

Limitations of this study are the routine use of throat swabs, known to have lower early viral loads than nasopharyngeal swabs, the incomplete testing of all subjectively chemosensory disturbed patients due to lack of consent, and the refusal of virus analyses by some patients. However, the case numbers are representative and sufficient for statements based on them.

## 5. Conclusions

Olfactory screening significantly increases the diagnostic accuracy in COVID-19 patients compared to subjective assessment of olfactory loss. Compared to other pathogens, the loss of smell in SARS-CoV2 is much more pronounced, while other cold symptoms differ only slightly between individual virus types. This might point to the importance of acute olfactory loss in the differential diagnosis of seasonal colds.

## Figures and Tables

**Table 1:** Subjective sudden smell and/or taste loss in patients tested positive or negative, respectively, for SARS-CoV-2.

			SARS-CoV-2		Total
			Negative	Positive	
Subjective smell and/or taste loss	No	*n*	1316	490	1806
		%	72.9	27.1	100
	Yes	*n*	177	137	314
		%	56.4	**43.6**	100
Total		*n*	1493	627	2120

**Table 2:** Psychophysically determined sudden smell loss in patients with subjective chemosensory loss tested positive or negative for SARS-CoV-2.

			SARS-CoV-2		Total
			Negative	Positive	
Psychophysically determined smell loss	No	*n*	61	13	74
		%	82.4	17.6	100
	Yes	*n*	64	100	164
		%	39.0	**61.0**	100
Total		*n*	125	113	238

**Table 3:** Psychophysically determined sudden taste loss in patients with subjective chemosensory loss tested positive or negative for SARS-CoV-2.

			SARS-CoV-2		Total
			Negative	Positive	
Psychophysically determined taste loss	No	*n*	91	86	177
		%	51.4	48.6	100
	Yes	*n*	34	27	61
		%	55.7	**44.3**	100
Total		*n*	125	113	238

## Data Availability

The data that support the findings of this study are available from the corresponding author upon request.
